# Supplementary material for: A Bibliometric Analysis on the Research Trend of Exercise and the Gut Microbiome
Source: Microorganisms. 2023 Mar 30;11(4):903. doi: 10.3390/microorganisms11040903 (PMC10141121; doi:10.3390/microorganisms11040903)
Supplement: Supplementary file 1 [file microorganisms-11-00903-s001.zip › microorganisms-2261934-supplementary.pdf]

Supplementary Material

A Bibliometric Analysis on the Research Trend of Exercise and the Gut Microbiome

Ruiyi Deng [1,†], Mopei Wang [2,†], Yahan Song [3,*] and Yanyan Shi [1,*]

1 Research Center of Clinical Epidemiology, Peking University Third Hospital, Beijing 100191, China
2 Department of Medical Oncology and Radiation Sickness, Peking University Third Hospital, Beijing 100191, China
3 Library, Peking University Third Hospital, Beijing 100191, China
* Correspondence: puh3_lib@bjmu.edu.cn (Y.S.); shiyanyan@bjmu.edu.cn (Y.S.); Tel.: +86-1561-1908-598 (Y.S.); +86-1561-1963-635 (Y.S.)
† These authors contributed equally to this work.

1 Search strategy

#1:

TS= ('Exercises' OR 'Physical Activity' OR 'Physical Activities' OR 'Physical Exercise' OR 'Physical Exercises' OR 'Acute Exercise' OR 'Acute Exercises' OR ' Aerobic Exercise' OR 'Aerobic Exercises' OR 'Exercise Training' OR 'Exercise Trainings' OR 'Remedial Exercise ' OR 'Remedial Exercises' OR 'Exercise Therapies' OR 'Rehabilitation Exercise' OR ' Rehabilitation Exercises' OR 'Human Physical Conditioning' OR 'Human Physical Training' OR 'Sport' OR 'Athletics' OR 'Athletic' OR 'Resistance Training' OR 'Strength Training' OR 'Endurance Training' OR 'High-Intensity Interval Training' OR 'High-Intensity Interval Trainings' OR 'High-Intensity Intermittent Exercise' OR 'High-Intensity Intermittent Exercises' OR 'Sprint Interval Training' OR 'Sprint Interval Trainings' OR 'Runnings' OR 'Running' OR 'Joggings' OR 'Jogging' OR 'Swimming' OR 'Walking' OR 'Ambulation' OR ' Marathons' OR 'Marathon' OR 'Ultramarathon Running' OR 'Marathon Running')

#2:

TS= ('Gastrointestinal Microbiome' OR 'Gastrointestinal Microbiomes' OR 'Gut Microbiome' OR 'Gut Microbiomes' OR 'Gut Microflora' OR 'Gut Microbiota' OR 'Gut Microbiotas' OR 'Gastrointestinal Flora' OR 'Gut Flora' OR 'gut microecology' OR 'Gastrointestinal Microbiota' OR 'Gastrointestinal Microbiotas' OR 'Gastrointestinal Microbial Community' OR 'Gastrointestinal Microbial Communities' OR 'Gastrointestinal Microflora' OR 'Gastric Microbiome' OR 'Gastric Microbiomes' OR 'Intestinal Microbiome' OR 'Intestinal Microbiomes' OR 'Intestinal Microbiota' OR 'Intestinal Microbiotas' OR 'Intestinal Microflora ' OR 'Intestinal Flora' OR 'Enteric Bacteria' OR 'intestinal microbial population' OR ' intestinal microecology' OR 'enteric microorganism' OR 'intestinal microorganism' OR ' Akkermansia' OR 'Bifidobacterium' OR 'Clostridia' OR 'Eubacterium' OR 'Firmicutes' OR 'Faecalibacterium' OR 'Lactobacillus' OR 'Ruminococcaceae' OR 'Streptococcus')

#3: #1 AND #2
